# The Applications of Nanotechnology in Crop Production

**DOI:** 10.3390/molecules26237070

**Published:** 2021-11-23

**Authors:** Chenxu Liu, Hui Zhou, Jie Zhou

**Affiliations:** 1 Department of Horticulture, Zhejiang University, Yuhangtang Road 866, Hangzhou 310058, China; chenxuliu@zju.edu.cn (C.L.); 3180100610@zju.edu.cn (H.Z.); 2 Key Laboratory of Horticultural Plants Growth, Development and Quality Improvement, Agricultural Ministry of China, Yuhangtang Road 866, Hangzhou 310058, China; 3 Shandong (Linyi) Institute of Modern Agriculture, Zhejiang University, Linyi 276000, China

**Keywords:** plants, nanobiotechnology, nanofertilizers, nanopesticides, nanosensors, nanotechnology

## Abstract

With the frequent occurrence of extreme climate, global agriculture is confronted with unprecedented challenges, including increased food demand and a decline in crop production. Nanotechnology is a promising way to boost crop production, enhance crop tolerance and decrease the environmental pollution. In this review, we summarize the recent findings regarding innovative nanotechnology in crop production, which could help us respond to agricultural challenges. Nanotechnology, which involves the use of nanomaterials as carriers, has a number of diverse applications in plant growth and crop production, including in nanofertilizers, nanopesticides, nanosensors and nanobiotechnology. The unique structures of nanomaterials such as high specific surface area, centralized distribution size and excellent biocompatibility facilitate the efficacy and stability of agro-chemicals. Besides, using appropriate nanomaterials in plant growth stages or stress conditions effectively promote plant growth and increase tolerance to stresses. Moreover, emerging nanotools and nanobiotechnology provide a new platform to monitor and modify crops at the molecular level.

## 1. Introduction

Nanotechnology is a prospective field with multiple applications across various areas of modern science, including physics, pharmacology chemistry, computer science, agriculture and engineering [1]. The distinct physical, chemical and biological properties of nanoparticles (NPs) give them the ability to modify typical chemicals and devices [2]. NPs are defined as natural and artificial materials with at least one dimension ranging from 1 nm to 100 nm and these materials can be organic, inorganic or polymeric compounds [3].

With the rapid growth of the population and deterioration of the environment, traditional agriculture is facing unprecedented challenges [3]. Fertilizers and pesticides have played pivotal roles in satisfying food production needs for decades [4]. However, excessive use of these chemicals also hinders sustainable agricultural development [5,6]. Increased use of nanotechnology could provide innovative solutions to improve sustainable agriculture, which would also fulfill food demands [7]. Current studies have shown that nanotechnology can be widely used to address various agricultural problems, such as excessive use of fertilizers and pesticides, and plant stress induced by extreme climate [8,9,10,11,12]. Besides, nanomaterials significantly promote plant growth, seed germination and stress tolerance [3]. Moreover, applications of nanotechnology also include plant growth status monitoring, rapid and simple genome modification and transgene expression in intact plant cells [13,14].

Currently, research involved in nanotechnology applied in agriculture has increased exponentially. However, few reviews integrate all aspects of nanotechnology used in crop production together, especially the emerging nanosensors and nanobiotechnology. In this review, we summarize the current research on nanotechnology in crop production, which helps us develop sustainable agriculture.

## 2. Nanofertilizers

**Figure 1:**
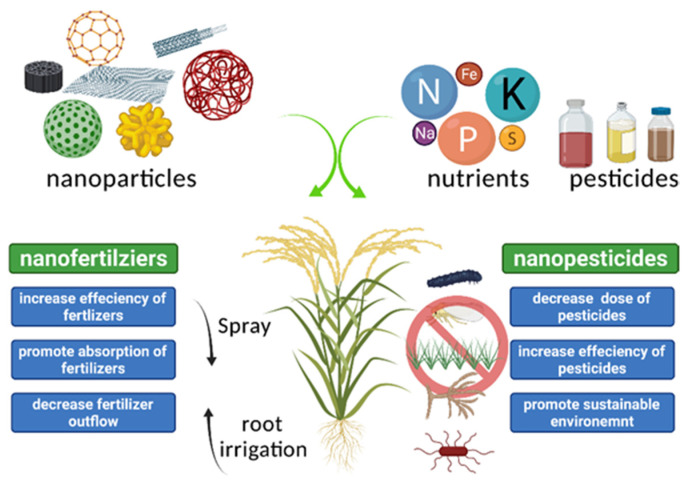
Simplified application model of nanofertilizers and nanopesticides. Fertilizers and pesticides are encapsulated by versatile nanoparticles. Nanofertilizers and nanopesticides can be applied by spraying or irrigation to increase the efficiency of nanochemicals, promote the absorption of fertilizers, decrease fertilizer outflow and pesticides doses and promote environmental sustainability.

Chemical fertilizers are indispensable for modern agricultural systems. However, the efficiency of synthetic chemical products has been decreasing for decades, accompanied by water pollution, soil contamination and greenhouse gas emissions [3]. Nanotechnology could pave the way for sustainable agriculture (Figure 1).

Nanofertilizers are mineral nutrients manufactured mainly by encapsulation with NPs and can be classified into macronutrients and micronutrients [4]. Macronutrients such as carbon (C), nitrogen (N), potassium (K), phosphorus (P), calcium (Ca), sulfur (S) and magnesium (Mg) have been encapsulated by different nanomaterials, to improve crop absorption of fertilizers and decrease fertilizer outflow [6,15,16,17,18,19,20]. The high specific surface area, high stability and excellent biocompatibility of NPs provide NP fertilizer composites with increased release efficiency [21]. For example, urea-hydroxyapatite (HA) NPs have exhibited great potential for prolonging the release time and reducing the consumption of nitrogen fertilizers. Urea obtains the advantages of NPs by interacting with amine and carbonyl groups of HA NPs [15]. Field trial data have shown that, compared with pure urea, nanohybrids of urea and HA increase agronomic nitrogen use efficiency by approximately 30% [15]. In addition, numerous studies have revealed that the high specific surface area and density of NPs endow nanohybrids with high reactivity [3,7,22]. The application of nanofertilizers has great promise for promoting fertilizer absorption and increasing crop yields [6]. Recent studies have reported that loading N, P and K into chitosan NPs increases the acquisition of N, P and K by 17.04%, 16.31% and 67.50%, respectively, compared to that of an untreated control in cultured coffee plants [16]. Sprayed on cotton, magnesium oxide (MgO) NPs significantly increased the seed cotton yield by 42.2% relative to the untreated control [19]. Furthermore, nanofertilizers also control the loss of fertilizers and decrease damage to the soil. Phosphate ions-loaded layered double hydroxide (LDH) significantly increased the soil pH value and decreased the soil absorption of P [17]. In conclusion, the nanohybrids mentioned above, with plentiful pores and less volume, facilitate nutrient uptake. Fertilizers encapsulated by NPs, especially porous NPs, are hardly damaged by environmental factors such as rain and wind while those compounds are easily transferred into plant cells. This feature prolongs the fertilizer release time and improves soil physical and chemical properties [3]. Specifically, traditional fertilizers chemically or physically interact with NPs such as HA or chitosan NPs, and these kinds of interactions help fertilizers escape degradation by environmental factors [15,16]. Moreover, chemical reactions of NPs and fertilizers decrease and homogenize the particles size of fertilizers. This process endows nanofertilizers with stability and high specific surface area, which significantly increases the efficiency of fertilizers [4].

Micronutrients such as iron (Fe), boron (B), manganese (Mn), copper (Cu), zinc (Zn) and molybdenum (Mo) have also been enclosed in nanomaterials such as chitosan, zinc oxide NPs (ZnO NPs), Cu NPs and Ca NPs, which improve the effective accessibility of micronutrients to plants [6,23,24,25]. These micronutrients play crucial roles in diverse plant physiological and chemical reactions, including photosynthesis, enzyme components and enzyme activators. Moreover, the application of nanofertilizers made of micronutrients promotes plant growth and increases yields [24,25]. For example, ZnO NPs fertilization of strawberry significantly increased fruit setting and the grain yield compared to strawberry fertilized with zinc sulfate (ZnSO_4_) [24]. B deficiency damages shoot growth and pollen germination. However, foliar application of calcium borate NPs (CaO_2_B_2_O_3_·10H_2_O NPs) to lettuce promoted the accumulation of B 1.54-fold and 3.95-fold in shoots and roots, respectively, compared with treatment using a nutrient solution in traditional B addition [25]. As mentioned above, extra fertilizing micronutrients can enhance the photosynthesis rate and antioxidant enzyme activity. These physiological processes lead to elevated dry weights, shoot lengths, root lengths and biomass. Hence, foliar or drench application of nanotype micronutrients significantly increases yields and promotes plant growth.

The size distribution of NPs is an effective parameter related to fertilization efficiency [26]. Interestingly, nanotype fertilizers all have decreased particles sizes and increased numbers of particles per unit, leading to high specific surface areas [4]. Increased interaction with leaves and roots enables better absorption of fertilizers by plants. In addition, the unique properties of NPs lead to enduring effects. NPs encapsulated fertilizers resist degradation by hydrolysis, photolysis, evaporation, microbial organism decomposition and weathering [3]. Furthermore, their porous structures and small size profiles may help NPs be transferred into cells by molecular transporters or ion channels, which activates signaling pathways related to phytohormones or other growth factors [21].

## 3. Nanopesticides

Nanoformulation or encapsulation of insecticides, herbicides, fungicides and bactericides with nanomaterials holds enormous potential for decreasing chemical pesticides doses, increasing crop production and promoting sustainable development [11,22]. Nanocarriers of nanopesticides include polymeric NPs (such as chitosan and solid lipids), inorganic nonmetallic NPs (such as silica NPs and nanoclays) and metallic NPs (such as Cu NPs and ZnO NPs) [27,28,29,30,31,32,33,34]. Several studies have shown that nanotype insecticides are more efficient at killing pests and less likely to cause side effects on humans [35]. For example, spinosad- and permethrin-loaded chitosan NPs applied to *Drosophila melanogaster* displayed reinforced bioavailability even at lower doses than free spinosad and free permethrin, and the nanocomposites caused decreased damages to humans and the ecological environment [27]. Upon encapsulation by NPs, the nanoinsecticide particles become smaller and more centralized, which endows them with stability and a slow release capacity. These properties increase the activity of insecticides and decrease their toxicity towards humans. In addition, the biological toxicity of highly concentrated NPs provides a unique pathway for directly inhibiting the growth of pests, bacteria and viruses. For instance, several nanoinsecticides take advantage of the toxicity of metallic NPs. Aluminum oxide (Al_2_O_3_) NPs exhibited great potential for eliminating *Sitophilus oryzae* on stored rice compared with bulk Al_2_O_3_ treatment [36]. Pheromones have been verified to be a promising and effective method to control pest populations [37]. Composites of nanocarriers and pheromones amplify the advantages of sex pheromones [38]. For example, methyl eugenol-loaded nanogels applied to guava orchards increased the number of trap catches compared with the control group containing only methyl eugenol [39].

Herbicides, which are widely used to clear weeds, have exhibited numerous side effects, including toxic effects on living organisms, water pollution and contamination of soil and air, since commercial production of these chemical compounds [40]. Encapsulation of herbicides by nanoparticles is a promising mean to decrease herbicide residues in environment and increase weed control efficiency [41]. Among various kinds of NPs, solid lipids are the most suitable nanocarriers for nanoherbicides due to their good chemical stability and simple metabolism [42]. For example, foliar application of metsulfuron methyl-loaded polysaccharide NPs to weeds growing in wheat significantly decreased the weed biomass compared with normal herbicide [43]. Moreover, the cytotoxicity of nanoherbicides and traditional herbicides was also detected by incubation with cells, and the results showed that herbicide-loaded NPs were less toxic than normal herbicides [43]. Another study of solid lipid NP-based nanoherbicides showed better release profiles and herbicidal activity than normal herbicides. Encapsulation of atrazine by solid lipid NPs significantly inhibited the growth of *Raphanus raphanistrum* (weed species) compared with the normal herbicide-treated group. Furthermore, the tested nanoherbicide concentration had no toxicity toward *Zea mays* [44].

As mentioned above, metallic NPs appear to have unique potential for producing nanobactericides and nanofungicides [45]. For example, compared with free-leaf extracts, nanobactericides composed of silver NPs (Ag NPs) and holy basil leaf extract showed increased inhibition of *Xanthomonas axonopodis* pv. *punicae* on pomegranate [28]. Experiments examining bacterial activity revealed that Cu NPs inhibited the growth of five bacteria, *Agrobacterium tumefaciens*, *Dickeya dadantii*, *Erwinia amylovora*, *Pectobacterium carotovorum* and *Pseudomonas savastanoi* pv. *Savastanoi* [46]. Furthermore, metallic NPs have the capacity to inhibit bacteria/fungi, and nonmetallic NPs can suppress plant diseases. For instance, chitosan NPs effectively controlled infection by *Xanthomonas campestris* in chili peppers compared with the untreated group [47].

Similarly, metallic NPs also inhibit fungi. For example, cobalt ferrite (CoFe_2_O_4_) NPs and nickel ferrite (NiFe_2_O_4_) NPs reduced the incidence of *Fusarium wilt* compared with that in untreated plants, and these NPs had no side effects on the growth of *Capsicum* plants [31]. Moreover, the complexes of NPs and fungicides have concentrated particle size distributions and large specific surface areas, which improve antifungal activity and prolong fungicide release times [48]. Several studies have reported that fungicides encapsulated by NPs performed superbly in controlling fungi [30,49]. Chitosan-hexaconazole NPs crosslinked with tripolyphosphate (TPP) enhanced the inhibition of the growth of *Ganoderma boninense* compared with pure hexaconazole [49].

In addition to bacteria and fungi, phytoviruses lead to tremendous crop production losses due to their rapid duplication, genomic diversity and dynamic evolution (Table 1) [50]. NPs have become promising management tools to prevent viral invasion in different ways, such as by interacting with nucleic acids, triggering plant immune responses and delivering RNA interference systems. Foliar application of carbon nanotubes to tobacco effectively suppressed symptoms of the *Tobacco mosaic virus* (TMV) relative to untreated control. The relative expression level of viral coat proteins decreased in carbon nanotubes (CNTs)-treated plants. The concentrations of salicylic acid and abscisic acid in CNTs-treated plants dramatically increased over those in the untreated group. These results show that CNTs inhibit TMV infection by hindering viral replication and movement [51]. A recent study reported that metallic NPs such as Ag NPs can interact with the coat protein and induce a plant immune response to inhibit infection by the *Tomato mosaic virus* (ToMV) and *Potato virus Y* (PVY). Infection of tomato by ToMV and PVY decreased compared to untreated control when sprayed with Ag NPs. Meanwhile, the total soluble protein (TSP) content and polyphenol oxidase (PPO) activity in tomatoes infected with ToMV significantly increased compared with the control [52].

## 4. Nanotechnology in Regulating Seed Germination, and Plant Growth

**Figure 2:**
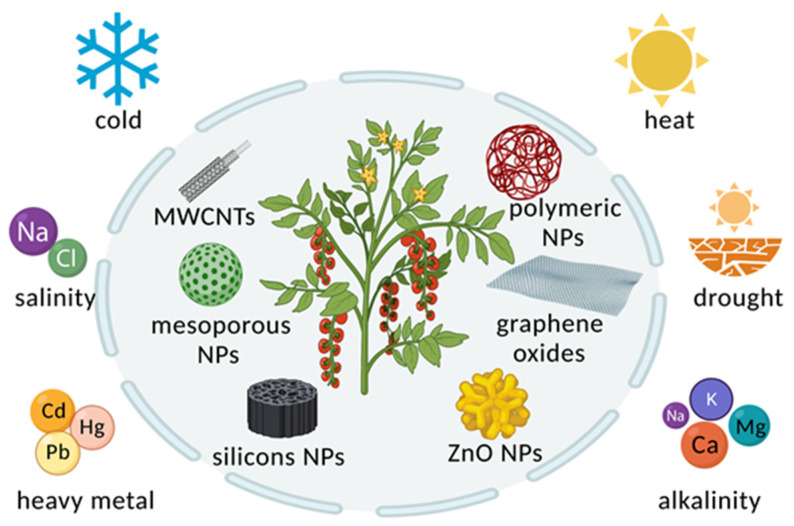
Overview of applications of nanoparticles (NPs) in stress tolerance. NPs, including multiwalled carbon nanotubes (MWCNTs), mesoporous NPs, silicon NPs, polymeric NPs, graphene oxides and zinc oxide (ZnO) NPs, have been successfully applied to help corps adapt to different stresses.

**Figure 3:**
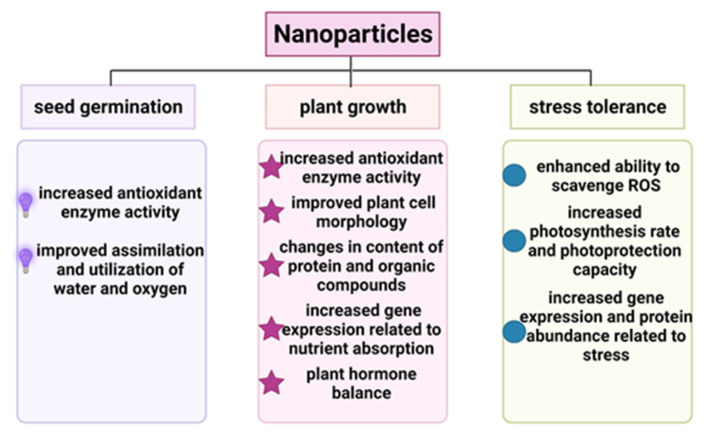
The mechanism of nanoparticles induced enhancement in seed germination, plant growth and stress tolerance.

Nanotechnology has been used in various aspects of agricultural production, such as seed germination and plant growth, to increase crop yields and quality (Figure 2 and Figure 3). Seed germination is a refined and fundamental biological process associated with environmental factors, genetic traits, and soil parameters. Recently, some studies have shown that NPs such as CNTs, silicon dioxide (SiO_2_) NPs, ZnO NPs, titanium dioxide (TiO_2_) NPs and even gold (Au) NPs have positive effects on seed germination in crop plants, including tomato, wheat, rice, pearl millet, soybean, barley and maize [12,53,54,55,56,57,58,59]. Seed germination is related to antioxidant enzyme activities and the contents or utilization rates of water and oxygen [60]. For example, Au NPs significantly increased the germination rate of pearl millet compared to that of untreated plants [54]. The seed germination rate of wheat treated with ZnO NPs was increased compared with that of the control group [61]. The two NPs mentioned above both have the ability to increase antioxidant enzyme activity. TiO_2_ NPs are beneficial to promoting seed germination, and exogenous treatment with TiO_2_ NPs enhances the seeds absorption of water and oxygen, leading to decreased germination time. For instance, tomato seeds soaked with TiO_2_ NPs exhibited a germination percentage increased by approximately 8% compared with the untreated control [59]. Another study has revealed that TiO_2_ NPs stimulate seed germination and dramatically decrease mean germination time in wheatgrass [57]. Moreover, nonmetallic NPs such as multiwalled carbon nanotubes (MWCNTs) can stimulate seed germination in different crops by increasing the seed water assimilation capability. Air-spraying MWCNTs on soybean, barley and corn seed successfully increased the seed germination rate by at least 25% compared with the untreated control. Further experiments revealed that MWCNTs penetrated the surface of the seed. Moreover, the relative gene expression of several water-channel-related genes in soybean, barley and corn seeds sprayed with MWCNTs increased significantly [12].

Additionally, NPs such as Ag NPs, ZnO NPs, TiO_2_ NPs, silica NPs and MWCNTs can promote the growth, photosynthesis and yield of many crop species, such as spinach, cotton, maize, soybean and barley [12,54,62,63,64,65]. NPs primarily accelerate plant growth by mediating crop antioxidant enzyme activity. For instance, ZnO NPs sprayed on cucumber improved the plant chlorophyll content and leaf fresh/dry weight. Antioxidant-related enzyme activities, such as superoxide dismutase (SOD) and catalase (CAT) activities, in the treated cucumber leaves all increased significantly compared with the untreated control [66]. In addition, NPs influence plant cell morphology and improve protein and organic compounds content of the cell [62]. Soil amended with silica NPs promoted the growth of maize, especially in terms of plant height and root length. Moreover, differences in plant morphology may be linked to the thickness of the cell wall [62]. Silica-NP-treated plants showed thicker cell walls and more silica bodies in root cells compared to the control plant. Meanwhile, the protein content in silica-NP-treated plants was higher than that in the bulk-silica-treated one. However, organic compounds such as phenols, aldehydes and ketones were less abundant in silica-NP-treated plants [62]. NPs tend to induce gene expression related to nutrient assimilation and growth regulation [12,60]. The bioinformatics helps researchers dig deeper for information [67]. The transcriptome of ZnO-NPs-treated seedlings revealed that several metal-accumulation-related genes such as *BASIC HELIX-LOOP-HELIX 38 (bHLH38)*, *bHLH39*, *bHLH100*, *ZINC TRANSPORTER 9 (ZIP9)* and *IRON-REGULATED TRANSPORTER 1 (IRT1)* were upregulated in seedlings treated with ZnO NPs compared with those treated with normal Zn ions [60]. NPs also have the potential to regulate plant hormone balance [68,69]. Foliar application of Ag NPs to two varieties of common bean (Bronco and Nebraska) induced gene expression related to the auxin signaling pathway, leading to a high content of auxin in plants [68].

Due to their structural and surface reactivity properties, NPs can induce intracellular oxidative stress and genetic damage, which can lead to reduced crop yields and physiological disorders when high concentrations of NPs are applied [55]. As mentioned above, metallic NPs always have side effects on organisms due to the toxicity of metal elements. Depending on this property, NPs can be a suitable resource for nanopesticides, but they are also likely to inhibit plant growth and development. Ag NPs at 500 mg/L significantly decreased the biomass of squash by 74% compared with the untreated control. In addition, squash cultured in Hoagland’s solution amended with 100 mg/L Cu NPs exhibited 93% reduction in biomass relative to the untreated control [70]. Therefore, we must determine the safest doses of various NPs for different crop species. 

## 5. Nanotechnology in Mediating Abiotic Stress Tolerance

As sessile organisms, plants are readily exposed to abiotic stresses such as cold, heat, drought, salinity, soil alkalization and heavy-metal contamination, which strongly affect food production and safety [71,72]. Several studies have indicated that different nanomaterials, including ZnO NPs, TiO_2_ NPs, Fe_2_O_3_ NPs, silicon (Si) NPs, nanoceria, graphene oxides and MWCNTs, reduce the deleterious effects of abiotic stress on crop plant species such as potato, barley, alfalfa, sugar beet, flax, maize, *Arabidopsis thaliana* and rice [73,74,75,76,77,78,79,80,81].

NPs enhance plant tolerance to abiotic stress mostly by scavenging ROS and increasing antioxidant enzyme activities [82]. Recent research has shown that graphene NPs increase the alfalfa tolerance of alkaline conditions, specifically by improving antioxidant enzyme activities and increasing the fresh weight, dry weight and seedling root length [80]. Ce ions can react with hydroxyl radicals, superoxide anions and hydrogen peroxide to generate harmless substances such as oxygen, water and hydroxide ions. Polyacrylic acid nanoceria (PNCs) with a low ratio of Ce^3+^/Ce^4+^-reduced ROS levels in Arabidopsis thaliana leaves [81]. Another study revealed that MgO NPs alleviated lead (Pb) stress in *Daucus carota* by increasing the activities of SOD and CAT. Specifically, MgO NPs treatment increased the activities of SOD and CAT by 29% and 32%, respectively, under Pb stress relative to the untreated control. MgO NPs treatment also increased the level of polyamines, which play important roles in plant growth and development [83]. Chitosan-polyvinyl alcohol (Cs-PVA) hydrogels and Cu NPs combined treatment increased the expression of SOD compared with the control in tomatoes under salt stress [84].

Additionally, NPs can elevate plant tolerance to stress by increasing the photosynthesis rate and photoprotection [82]. For instance, the chilling stress-induced reduction in the photosynthesis rate in sugarcane was relieved by multiple NPs, including SiO_2_ NPs, ZnO NPs, selenium (Se) NPs and graphene nanoribbons (GNRs). Compared with the untreated control, foliar application of SiO_2_ NPs increased the maximum photochemical efficiency of PSII (Fv/Fm), maximum photooxidizable P700 (Pm) and photosynthesis rate (Pn) by 16.7%, 21.3% and 74.5%, respectively. The other three NPs listed above also elevated these parameters, especially Pn, which increased by at least 47.2% relative to the control group [85]. Pearl millet seeds were soaked in a Ag NPs solution before priming, and then parameters related to photosynthesis in seedlings under salt stress were detected. The results revealed that the photosynthesis rate, transpiration rate and stomatal conductance of treated plants increased by 148%, 109% and 62% relative to the untreated control, respectively [86].

Besides, NPs induce genes expression associated with stress and increase the abundance of multiple proteins in plants under abiotic stress [87,88]. For example, several metal-based NPs increased the drought tolerance of soybean [88]. The expression of three stress-related transcription factors, *GmWRKY27*, *GmMYB117* and *GmMYB174*, in leaves treated with Fe NPs was 8-fold, 6-fold and 4-fold that in the control group under drought stress [88]. Label-free proteomics were used to reveal differences in the protein abundance of wheat roots treated with Fe NPs under drought conditions. The abundance of the Rubisco protein in plants exposed to Fe NPs was 3-fold that in untreated plants [87]. A few metallic NPs, such as Al_2_O_3_ NPs, ZnO NPs and Ag NPs, were used on soybean to relieve flooding stress. Among these NPs, Al_2_O_3_ NPs performed better than the others in promoting plant growth and decreasing sensitivity to stress. The proteomics of soybean seedlings under flooding revealed that the protein abundance related to protein synthesis, glycolysis and lipid development was increased upon Al_2_O_3_ NPs exposure [89]. Hence, the use of nanomaterials constitutes an effective and environmentally friendly method to enhance plant tolerance to abiotic stress. However, the toxicity of NPs to plants or the environment still needs to be considered before using it.

## 6. Nanosensors Used to Monitor Living Plants

**Figure 4:**
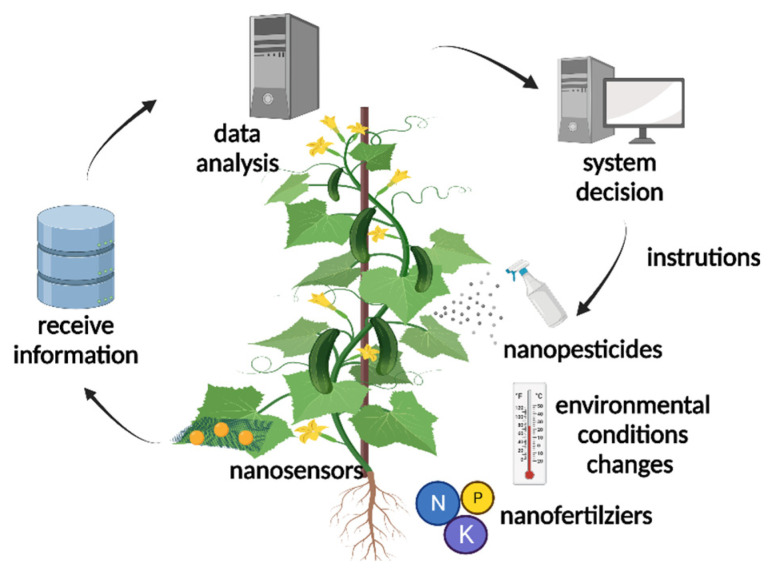
Applications of nanosensors in crops. Nanosensors used in plant monitoring include several aspects. Firstly, physiological or environmental parameters of plants are monitored by nanosensors. These data are delivered to electronic equipment, including a smartphone or laptop, immediately. Secondly, computer system analyzes data and provides instructions. Finally, the cultivation system or administrators adjust environment conditions and take measures according to instructions.

Agricultural applications of nanosensors involve nutrient management, growth monitoring, pest and disease assessment, detection of soil conditions, food production and plant hormone detection [90]. Nanosensors constitute a new platform for monitoring plant growth and development, which achieves nondestructive and accurate monitoring, and can be applied to individual plants in real time (Figure 4) [3]. Common nanosensor detection techniques include fluorescence resonance energy transfer (FRET), surface enhanced Raman scattering (SERS), corona-phase molecular recognition and common nanosensors themselves include electrochemical nanosensors and piezoelectric nanosensors [14,91,92,93,94,95].

Nanosensors used in living plants can be divided into several varieties, including plant signal, growth and stress sensors. First, multiple plant signaling molecules, including gas, electrical, phytohormone and chemical signals, can be detected by nanosensors [96,97,98,99]. Gas signals such as oxygen and nitric oxide (NO) are important internal plant signals in response to abiotic or biotic stress [100,101]. A fluorescent ratiometric single-walled carbon nanotubes (SWCNTs) sensor for NO detection is a nanosensor based on a single-molecule detection technique. The response of SWCNTs sensors in leaves was similar to that in in vitro tests, which indicated that this nanosensor has the capacity to deal with complex environments [101]. In addition, electrical/Ca^2+^ signaling molecules are fundamental signaling molecules in organisms and are associated with multiple abiotic and biotic stresses. Several indicators such as YC3.6, GCaMP and GCaMP-type low-affinity red fluorescent genetically encoded Ca^2+^ indicators for optical imaging (LAR-GECO), based on the FRET technique, provide visible, rapid and high affinity ways to detect transient Ca^2+^ [102,103,104]. Besides, a needle transistor-based sensor constituted by SWCNTs selectively detects Ca^2+^ in living cells, although this kind of sensor still does not function in plants [105]. Phytohormones are the most fundamental plant growth regulators involved in all life cycles of plants. Current studies associated with nanosensors of phytohormones include strigolactone, ethylene, jasmonic acid, abscisic acid and methyl salicylate (a ramification of salicylic acid) [99,106,107,108,109]. Researchers have developed a fluorescence turn-on probe named Yoshimulactone Green (YLG). YLG competes with synthetic or natural strigolactone to bind with the receptor of strigolactone, and these reactions produce detectable fluorescent products [99]. Chemical signals in plants, such as volatile organic compounds (VOCs), are always connected to food quality or plant abiotic/biotic stresses [110,111]. Sensing of these chemical signals is useful for predicting shelf life, decreasing loss and enhancing stress tolerance. As a basic fruit ripening indicator, malic acid has great potential as a target of nanosensors. A recent study showed that NADP-malate dehydrogenase (malic enzyme) is covalently immobilized on MWCNTs, and differential pulse voltammetry (DPV) is used to detect the concentration of malic acid in tomatoes. The malic acid nanosensor is rapid, reliable and sensitive in tests [110]. In addition, near-infrared fluorescent SWCNTs are selective sensors of hydrogen peroxide, which is a basic stress-related plant signaling molecule; thus, hydrogen peroxide nanosensors could help monitor remote and localized plant situations [96].

Sucrose and glucose are basic energy resources for plant growth, and detection methods for these chemicals have been upgraded in recent decades. The FRET technique is used frequently in monitoring the flux of sucrose and glucose [112,113]. Moreover, wearable nanosensors for use in people’s daily lives have developed rapidly and plentifully. Plant wearable nanosensors have also emerged for monitoring plant growth parameters. Water transportation and distribution are significant biological progresses in plant growth and development. A flexible electronic sensing device was developed to continuously monitor water transportation, sap flow and nutrient distribution. The application of this nanosensor to watermelon revealed a day/night shift in water distribution between fruits and leaves [114]. Before this nanosensor was reported, wearable nanosnesors made of vapor-printed polymer electrodes reliably detected deep tissue damage induced by dehydration and ultraviolet A radiation [115]. Another significant wearable nanosenor is a polyaniline (PANI)-coated MWCNTs ammonia sensor with high sensitivity, reliability and a fast response time in ammonia detection [116].

Nanosensors for plant disease diagnosis are significant for monitoring plant health and taking immediate defensive actions [117]. The accuracy, convenience and detection conditions of traditional detection tools limit their development [14]. Portable, economical and accurate nanosensors assist researchers in recognizing plant pathogens in a timely manner [117,118]. Targets of plant disease recognized by nanosensors include DNA, protein and VOCs [119]. For example, compared with the normal polymerase chain reaction method, the SERS-recombinase polymerase amplification (RPA) method was more sensitive and had a lower limit of detection in recognizing three important plant pathogens, *Botrytis cinerea*, *Pseudomonas syringae* and *Fusarium oxysporum* [120]. The mechanism of binding between antigens and antibodies is widely used in nanosensors to detect plant pathogens. The fluorescence of cadmium-telluride quantum dots (CdTe-QDs) conjugated with an antibody against *Citrus tristeza virus* (CTV) was activated by binding with CTV, and the fluorescence was quenched by competitive binding with the coat protein of CTV [121]. *p*-Ethylguaiacol is a typical VOC of strawberry that is produced due to infection by *Phytophthora cactorum*. A recent study showed that metal oxide NPs such as TiO_2_ or stannic oxide (SnO_2_) on screen-printed carbon (SP) electrodes detect *p*-ethylguaiacol sensitively and accurately [122]. In conclusion, nanosensors help administrators monitor plant health at the molecular level, which dramatically increases efficiency of plant management. However, the stability of these nanosensors still needs to be considered more when leveraged in agricultural systems. Moreover, are the sensitivity and reliability of nanosensors sufficient for use in agricultural production? We have confidence that these problems will be resolved in the future.

## 7. Nanobiotechnology in Genome Modification

In addition to widely used nanosensors, nanobiotechnology, especially nanomaterial-assisted biomolecule (such as DNA and RNA) transfer, is a promising research field [3]. Nanomaterial-assisted biomolecule transfer is involved in transgene expression, genome editing, gene silencing [8,123,124]. The physical and chemical properties of the plant cell wall hinder the transformation of biomolecules into plant cells. Pollen, as a typical plant tissue with a chemically inert cell wall, is an ideal target for transient gene expression. Imidazolium-coated SWCNTs were used to assist the transfer of plasmid DNA encoding green fluorescent protein (GFP) into oil palm pollen. The efficiency of both the delivery and activity of GFP was high [123]. Moreover, using chitosan-coated SWCNTs, a DNA plasmid was transformed into chloroplasts. This experiment comprised several plants species including *Eruca sativa*, *Nasturtium officinale*, *Nicotiana tabacum* and *Spinacia oleracea*, and carriers exhibited high transient expression levels [13]. In addition to transgene expression, studies associated with nanomaterial-based gene silencing and genome editing have dramatically increased in recent years. Nanomaterial-based specific delivery of genetically engineered plasmids provides innovative approaches for rapidly modifying the genomes of plants [8]. For instance, a recent study showed that conjugates of DNA and CNTs were successfully transferred into multiple plant species including tobacco, arugula, cotton and wheat [125]. The siRNA delivery platform mediated by CNTs exhibited high silencing efficiency in plant cells, and the NP-based delivery platform showed effective intracellular transferable capacity [126]. Polyethylenimine-coated Au NPs (PEI-AuNPs) successfully delivered siRNA into intact plant cells, and the target gene expression decreased by at least 76% [124]. The increasingly popular nanobiotechnology field provides tremendous opportunities for scientists to optimize systems for plant transformation. However, the stability of nanobiotechnology-assisted genome modification needs more study. Moreover, this kind of genome modification would induce problems for other species. There is still a need for more research to complete this project.

## 8. Conclusions

Nanotechnology applications in agriculture exhibit great potential for improving the environment and increasing the production and quality of crop plants [90]. In this review, we summarize current research involved in nanotechnology applied to crop production, which includes nanofertilizers, nanopesticides and nanomaterials used in enhancing plant growth, seed germination and stress tolerance, nanosensors and nanobiotechnology. However, in addition to the positive aspects of nanotechnology, there are still many gaps that exist between laboratory research and agricultural production. For instance, NPs are toxic but also beneficial when applied to crops, and various NPs concentrations need further study in distinct crop species. Besides, we also should find an economical point of application of nanomaterials that balance crop production and environmental protection. Moreover, how can these nanosensors be leveraged in agricultural systems? Are the sensitivity and reliability of nanosensors sufficient for use in agricultural production? Additionally, are there any differences between plants transformed via NPs and plants transformed via traditional methods? Nevertheless, the growing prospects of nanotechnology still increase confidence in the ability to meet the food demands of humans.

## Figures and Tables

**Table 1:** Effects of nanoparticles (NPs) on crop protection.

NPs	Type	Original Pesticide	Concentration	Target	Reference
Chitosan	Insecticide	Spinosad/permethrin	10 mg/L	*Drosophila melanogaster*	[27]
Zinc oxide (ZnO)	Insecticide	*Aspergillus niger*	20 mg/L	*Holotrichia* sp.	[34]
Aluminium oxide (Al_2_O_3_)	Insecticide	—	2 g/kg	*Sitophilus oryzae* L.	[36]
Nanogel	Insecticide	Methyl eugenol	12 mg/mL	*Bactrocera dorsalis*	[39]
Nanogel	Herbicide	Savory essential oil	15 mL/L	weeds	[29]
Polysaccharide	Herbicide	Metsulfuron methyl	0.5 g/L	*Chenopodium album*	[43]
Solid lipid	Herbicide	Atrazine and simazine	0.3 kg/ha	*Raphanus raphanistrum*	[44]
Silver (Ag)	Bactericide	Leaf extracts (holy basil)	15 mM	*Xanthomonas axonopodis* pv. *punicae*	[28]
Copper (Cu)	Bactericide	—	240 mg/L	*Agrobacterium tumefaciens*, *Dickeya dadantii*, *Erwinia amylovora*, *Pectobacterium carotovorum* and *Pseudomonas savastanoi* pv. *Savastanoi*	[46]
Chitosan	Bactericide	Streptomycin sulfate	1 mg/mL	*Xanthomonas campestris*	[47]
Cobalt ferrite (CoFe_2_O_4_) and Nickel ferrite (NiFe_2_O_4_)	Fungicide	—	500 mg/L	*Fusarium oxysporum*, *Colletotrichum gloeosporioides* and *Dematophora necatrix*	[31]
Chitosan	Fungicide	Hexaconazole	10 ug/L	*Ganoderma boninense*	[49]
Cu	Fungicide	—	0.5 mg/mL	*Fusarium solani* and *Fusarium oxysporum*	[30]
Carbon nanotubes (CNTs)	Antiviral-pesticide	—	200 mg/L	*Tobacco mosaic virus* (TMV)	[51]
Ag	Antiviral-pesticide	—	50 mg/L	*Tomato mosaic virus* (ToMV) and *Potato virus Y* (PVY)	[52]

## Abbreviations

Nanoparticles: NPs; Carbon: C; Nitrogen: N; Potassium: K; Phosphorus: P; Calcium: Ca; Sulfur: S; Magnesium: Mg; Hydroxyapatite: HA; Magnesium oxide: MgO; Layered double hydroxide: LDH; Iron: Fe; Boron: B; Manganese: Mn; Copper: Cu; Zinc: Zn; Molybdenum: Mo; Zinc oxide: ZnO; Magnetite: Fe_2_O_3_; Manganese zinc ferrite: Mn_0.5_Zn_0.5_Fe_2_O_4_; Zinc sulfate: ZnSO_4_; Aluminium oxide: Al_2_O_3_; Silver: Ag; Cobalt ferrite: CoFe_2_O_4_; Nickel ferrite: NiFe_2_O_4_; Tripolyphosphate: TPP; *Tobacco mosaic virus*: TMV; Carbon nanotubes: CNTs; *Tomato mosaic virus*: ToMV; *Potato virus Y*: PVY; Total soluble protein: TSP; Polyphenol oxidase: PPO; Silicon dioxide: SiO_2_; Titanium dioxide: TiO_2_; Gold: Au; Multiwalled carbon nanotubes: MWCNTs; Superoxide dismutase: SOD; Catalase: CAT; *BASIC HELIX-LOOP-HELIX 38*:*bHLH38*; *ZINC TRANSPORTER 9*:*ZIP9*; *IRON-REGULATED TRANSPORTER 1*: *IRT1*; Silicon: Si; Polyacrylic acid nanoceria: PNCs; Lead: Pb; Chitosan-polyvinyl alcohol: Cs-PVA; Selenium: Se; Graphene nanoribbons: GNRs; PSII: Fv/Fm; Maximum photooxidizable P700: Pm; Photosynthesis rate: Pn; Fluorescence resonance energy transfer: FRET; Surface enhanced Raman scattering: SERS; Nitric oxide: NO; Single-walled carbon nanotubes: SWCNTs; Low-affinity red fluorescent genetically encoded Ca^2+^ indicators for optical imaging: LAR-GECO; Yoshimulactone Green: YLG; Volatile organic compounds: VOCs; NADP-malate dehydrogenase: malic enzyme; Differential pulse voltammetry: DPV; Polyaniline: PANI; Recombinase polymerase amplification: RPA; Cadmium-telluride quantum dots: CdTe-QDs; *Citrus tristeza virus*: CTV; Stannic oxide: SnO_2_; Screen-printed carbon: SP; Green fluorescent protein: GFP; Polyethylenimine: PEI
